# Natural Antioxidants from Seeds and Their Application in Meat Products

**DOI:** 10.3390/antiox9090815

**Published:** 2020-09-01

**Authors:** Paulo E. S. Munekata, Beatriz Gullón, Mirian Pateiro, Igor Tomasevic, Ruben Domínguez, José M. Lorenzo

**Affiliations:** 1 Centro Tecnológico de la Carne de Galicia, rúa Galicia no. 4, Parque Tecnológico de Galicia, San Cibrao das Viñas, 32900 Ourense, Spain; paulosichetti@ceteca.net (P.E.S.M.); mirianpateiro@ceteca.net (M.P.); 2 Department of Chemical Engineering, Faculty of Science, University of Vigo (Campus Ourense), As Lagoas, 32004 Ourense, Spain; bgullon@uvigo.es (B.G.); rubendominguez@ceteca.net (R.D.); 3 Department of Animal Source Food Technology, Faculty of Agriculture, Nemanjina 6, University of Belgrade, 11080 Belgrade, Serbia; tbigor@agrif.bg.ac.rs; 4 Área de Tecnología de los Alimentos, Facultad de Ciencias de Ourense, Universidad de Vigo, 32004 Ourense, Spain

**Keywords:** phenolic compounds, catechin, quality, myoglobin, lipid oxidation, rancidity

## Abstract

The use of synthetic antioxidants in the food industry has raised important questions about the effects of prolonged consumption on human health. On top of that, the consumption of meat products has been changing due to the awareness generated by health-related organizations. In this sense, exploring strategies to develop and produce healthier meat products has become a paramount concern. Several studies explored the composition of several seeds to characterize and explore the compounds with antioxidant activity, which are mainly composed of polyphenols. The use of antioxidant extracts in meat products has shown important results to delay the oxidative reactions in meat products derived from the processing and storage of meat products. Moreover, these extracts can also replace synthetic antioxidants and preserve the quality of meat products. Therefore, the aims of this review are first, to present the sources and compounds with antioxidant activity in seeds, and second, to discuss their protective effect against oxidative reactions in meat products.

## 1. Introduction

Oxidative reactions can drastically affect the quality and shelf life of meat and meat products [1]. The exposure to UV radiation, atmospheric oxygen, and intermediate radical species play a central role in the initial and progression of oxidative reactions that affect meat pigments, unsaturated fatty acids, proteins, and other components of muscle foods [2,3]. Once these components are oxidized, the formation and eventual decomposition of unstable intermediate radicals and accumulation of products lead to changes in the physicochemical, chemical, and sensory properties of meat and meat products [2,4,5].

Preventing or delaying the oxidative reactions in components of meat products can be carried out by adding antioxidants [6,7,8,9,10]. These compounds are defined as molecules (at low concentrations) that can protect or slow the oxidation of other molecules/substrates [11]. Antioxidants can act by different mechanisms to interrupt the formation of radicals and form stable products, to some extent, and preserve the quality and prolong the shelf life of muscle foods [12].

Although the food antioxidants are regulated and considered “safe” (if the daily intake is lower than that indicated by health and food authorities), an important share of meat consumers is willing to consume food products with “more natural” ingredients [13,14,15]. The scenario was generated by the potential risk associated with the consumption of synthetic antioxidants and the development of diseases such as cancer [2]. Consequently, this condition has forced the professionals and researchers of the meat area to search and select the most appropriate options to meet this trend [16,17,18,19,20,21]. The antioxidants found in natural sources fit in this trend, especially polyphenols found in seeds.

Plant seeds have been consumed since ancient times due to their nutritional value, especially for their macro- and micro-nutrients as well as their polyphenols with antioxidant activity in recent times [22,23]. Polyphenols are compounds with a di- or trihydroxyphenyl group that are directly involved in the stabilization of reactive species by donating a hydrogen atom or transferring an electron [24]. Moreover, these phenolic compounds can be mainly found in the coats (hull, husk, or skin, for instance) covering the cotyledon(s) of seeds such as reported for lentil, pea, beans, peanuts, and pistachio [25,26,27] and can be separated from the seed matrix by extraction with appropriate solvent [28]. It is also relevant to mention that some natural extracts rich in polyphenols (such as those obtained from *Rosmarinus officinalis* L., grape seed and skin, and olive pulp) are generally recognized as safe (GRAS) can be applied in food as antioxidants [16,29,30,31,32]. Due to the potential of natural extracts for the development of healthier muscle foods, this review aims to discuss the potential sources of polyphenols among seeds and their application in meat products.

## 2. Phenolic Compounds Found in Seeds

The characterization of phenolic compounds found in seeds reveals the variety of compounds that can be explored to improve the antioxidant capacity of meat products. Seeds rich in polyphenols can be found in several groups of vegetables that include cereals, pseudo-cereals, fruits, pulses, nuts, cruciferous vegetables, and other sources Table 1.

Regarding the cereal group, a relevant source of polyphenols is black rice (*Oryza sativa L.*). This ancient food was prized as the “forbidden rice” in China due to its historical importance in the diet of the Chinese Emperor [33]. Regarding the phenolic profile, cyanidin-3-glucoside, ferulic acid, isoferulic acid, *p*-coumaric acid, peonidin-3-glucoside, and vanillic acid were indicated as main compounds [33,34]. In the case of pseudo-cereals, buckwheat (*Fagopyrum esculentum* Moench), especially the hull, is a rich source of nutrients and functional components that was first documented in the Balkans and now is largely cultivated in several countries around the world [35]. The phenolic profile of buckwheat is composed of quercetin-3-rutinoside, caffeic acid-pentoside, 1-*O*-caffeoyl-6-*O*-rhamnopyranosyl-glycopyranoside, epiafzelechin–epicatechin dimer, epicatechin-gallate, hyperin, orientin and isoorientin, vitexin and isovitexin, and procyanidin trimers [35,36]. Likewise, chia (*Salvia hispanica* L.) seeds are a relevant source of nutrients (especially polyunsaturated fatty acids) originated from northern Guatemala and southern Mexico regions [37]. Caffeic acid, protocatechuic acid, rosmarinic acid, salicylic acid, daidzein, myricetin, and quercetin can be cited as relevant polyphneols found in chia seed [37,38].

In relation to fruit, cacao (*Theobroma cacao* L.) seeds are not only a crop for the production of the widely appreciated chocolate and cocoa powder but also an important source of polyphenols [39]. In relation to the main phenolic compounds found in cocoa beans husk, recent studies indicated the presence of epicatechin and catechin as the main compounds [39,40]. In a similar way, grape (*Vitis vinífera* L.) seed is a byproduct generated from the processing of grape [41]. Its phenolic composition comprises proanthocyanidins with different degrees of polymerizations as well as the monomers epicatechin gallate and (epi)catechin [42,43]. Guarana (*Paullinia cupana*) is a plant originally found in the Amazon forest that produces fruits that are consumed for its appreciated gastronomic uses and stimulatory effect [44]. The presence of tyrosols, proanthocyanidins of different degrees of polymerization (dimes, trimers, and tetramers), and (epi)catechin were indicated as main phenolic compounds [44,45,46].

In the category of pulses, lentil (*Lens culinaris*) is known for the high nutritional value, prebiotic potential, and low phytic acid content [55]. Moreover, lentil also contains phenolic compounds: quercetin-*O*-pentoside, catechin glucoside, prodelphinidin dimers, and procyanidin dimers [47]. In the case of nuts, peanut (*Arachis hypogaea* L.) and pistachio (*Pistacia vera* L.) can be cited as sources of polyphenols. Peanuts are widely consumed as raw or processed products such as snacks and butter, which contains co-enzyme Q10 and essential amino acids (such as arginine) [56]. The peanuts are also rich in phenolic compounds, especially the skin: protocatechuic acid, di-*p*-coumaroyltartaric acid, *p*-coumaroylsinapoyltartaric acid, catechin and proanthocyanidins with different degrees of polymerization (from dimers to nonamers) [48,49]. Pistachio can be considered as a nut with many nutrients (especially unsaturated fatty acids) that can assist in the prevention of cardiovascular diseases [26,57]. Along with macro and micronutrients, pistachio seed hulls are rich sources of phenolic compounds such as gallic acid, catechin, isoquercetin, myricetin-3-glucoside, naringenin, and quercetin-3-glucuronide [50,51].

From the cruciferous group, mustard (*Sinapis alba*) seeds are interesting sources of polyphenols. Mustard seeds are largely used as a condiment in food preparations [58]. A recent study indicated that the phenolic profile of mustard seeds hulls comprises sinapic acid and its derivatives sinapine and sinapoyl glucose [52]. Other relevant sources of polyphenols among seeds are acorn (*Quercus* spp.) and *Euryale ferox*. Acorn is the seed of the oak tree found in Europe, Asia, and North America [53,59]. The main phenolic compounds found in acorn are trigalloyl-hexahydrodiphenoyl-glucose, ellagic acid, and valoneic acid dilactone [53]. Finally, *Euryale ferox* is a plant used in traditional medicine to treat leukorrhea, hernia, and pyodermas in the Chinese traditional medicine [60]. The seeds of this plant contain phenolic compounds such as gallic acid, catechin, and rutin [54].

## 3. Influence of Extraction Conditions in the Polyphenol Content and Antioxidant Activity of Seed Extracts

**Figure 1:**
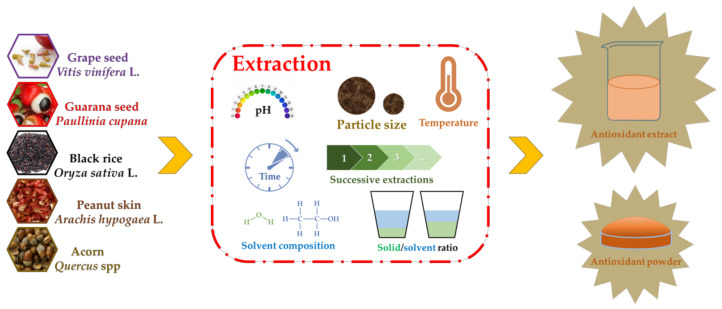
Factors affecting the extraction of phenolic compounds from seeds.

In order to use seed polyphenols in meat products, understanding the influence of the extraction technology and variables in phenolic content and antioxidant activity are of great value to define the most efficient conditions [61,62,63,64]. Figure 1 indicates the main factors associated with the extraction of phenolic compounds with potential applications in meat products.

Table 2 summarizes selected studies about the influence of extraction conditions in the phenolic content of seed extract. In the case of cereals and pseudo-cereals, a study using the conventional extraction approach in black rice revealed the influence of extraction time and temperature as well as solid/solvent ratio [65]. Performing the extraction at the optimum conditions, the authors reported a TPC of 520.17 mg/100 g and a correspondingly high level of antioxidant activity (46.5% of DPPH radical inhibition). In addition to the conventional extraction approach, the use of ultrasound can also be applied to obtain seed extracts rich in polyphenols from black rice [66]. Using this technology, the authors studied the influence of temperature, pH, solvent, extraction time, and temperature and indicated that the highest polyphenol extraction yield was 2124.98 mg/100 g.

In a similar way, the effect of solvent composition, the number of extractions, and extraction time were evaluated for the extraction of polyphenols from chia seeds [68]. According to the authors, an increase of 42% in the recovery of polyphenols was obtained by optimizing the solvent composition, the number of cycles, and extraction time. In the case of buckwheat, a recent experiment explored the influence of microwave irradiation as well as temperature and solvent composition in the extraction of polyphenols [67]. This technology increased the extraction of polyphenols in comparison to non-irradiated extraction (2.0–18.5 vs 1.4–9.2 mg/g, respectively) in comparison to conventional extraction, regardless of solvent composition or extraction temperature.

In the group of seeds obtained from fruits, Okiyama et al. studied the influence of extraction time and temperature in the extraction of polyphenols from cocoa bean shells using the pressurized liquid extraction approach [69]. The authors indicated that the flavonol content of extracts was enhanced by increasing temperature to 90 °C and time to 50 min. Accordingly, the antioxidant activity of the extract was also improved by increasing temperature to 90 °C as quantified using the 2,2-diphenyl-1-picrylhydrazyl (DPPH radical) assay (64–65 μmol TE/g) and for Ferric reducing ability of plasma (FRAP) assay (72–84 μmol TE/g), regardless of extraction time.

The effect of extraction conditions in the content of polyphenols in grape seed extracts was also evaluated in recent studies. For instance, Da Porto, Porretto, and Decorti indicated that defatting grape seeds prior to polyphenol extraction is a crucial factor to improve the yield of polyphenols in the extract. According to the authors, the highest polyphenol extraction yield and antioxidant activity were obtained using ultrasound in the defatting step and Soxhlet for the extraction of polyphenols [70]. In a related experiment with grape seeds, the efficiency of microwave irradiation was evaluated [71]. In this case, the authors indicated that the optimization of solvent composition, solid/solvent ratio, extraction temperature, and time were decisive to increase the content of polyphenols in the extract.

In the experiment carried out by Santana and Macedo [72], the polyphenols from guarana seed were extracted using the conventional extraction approach (cold and hot) and by the enzyme-assisted method. According to the authors, particle size and solvent composition played a central role to improve the extraction of polyphenols, particularly using particles with 1680 μm in diameter and 50% ethanol solution for cold (room temperature) conventional extraction; In the case of hot conventional extraction, increasing extraction time to 6 h was associated with a major effect on the polyphenol content of guarana extracts than increasing temperature from 40 to 60 °C. Additionally, the authors also evaluated the use of enzymes (pectinase and celulase) and temperature to enhance the polyphenol content in the guarana extract. The highest yield was obtained using pectinase at 50 °C. Marques et al. [73] studied the influence of pressure, temperature, co-solvent composition, and time during the extraction of polyphenols using super-critical CO_2_. The authors indicated that the content of polyphenols in the extract was affected by all tested variables.

In the category of nuts, the extraction of polyphenols from peanuts was optimized in terms of solvent composition, extraction time, and temperature in a recent study [74]. In this experiment, the temperature and solvent composition played a major role than extraction time to improve the extraction of polyphenols from peanut skin. The antioxidant activity of the extracts was also influenced by extraction conditions but the optimum values were lower temperatures than those reported for optimum polyphenol extraction (61.4 °C and 26.6 °C vs 71.6 °C for polyphenol, DPPH, and 2,2’-azino-bis-(3-ethyl-benzothiazoline-6-sulphonic acid (ABTS), respectively). A related experiment reported the effect of sub-critical water on the extraction of phenolic compounds from peanut skin [75]. According to the authors, the extraction yield was improved by increasing temperature and solvent flow (220 °C and 7 g/min, respectively), whereas 60.5% ethanol solution was indicated as the optimum solvent composition. Additionally, the extract obtained from optimum conditions was also associated with high antioxidant activity.

In a similar way, a recent experiment performed by Erşan et al. [76] explored the effect of sub-critical water and ultrasound-assisted extraction in the production of polyphenol-rich extracts from pistachio hulls. The authors indicated that sub-critical water extraction was gradually improved by enhancing temperature up to 170 °C, especially for gallic acid and penta-O-galloyl-β-D-glucose. Accordingly, the highest antioxidant activities were obtained from extracts using the highest temperatures (170 °C and 190 °C). Conversely, the extraction carried out with ultrasound produced an extract with higher polyphenolic content but lower antioxidant activity than those obtained from sub-critical water at 170 and 190 °C. The influence of solvent composition and temperature in the polyphenol content of acorn extracts was evaluated by Kyriakidou et al. [77]. The authors indicated that optimum extraction condition for polyphenols and antioxidant activity was obtained using a solvent composed of water, glycerol, and 2-hydroxypropyl-β-cyclodextrin (27:60:13) at 80 °C.

Additionally, it is important to consider the safety of the solvent to obtain the polyphenol-rich extracts from seeds in food. Ideally, water is a suitable solvent for the extraction of polyphenols since its use does not impose safety concerns [78]. However, the use of hydroethanolic solutions is of great value to increase the content of polyphenols and the antioxidant activity of extracts obtained from different seeds and extraction technologies [66,67,68,71,73,74,75]. Moreover, ethanol is an organic solvent that is considered safer than other organic solvents such as methanol [79]. In line with the safety concern of extraction solvent, the studies discussed below reported the effect of extracts obtained using water or a hydroethanolic solution. In the case of commercial extracts, the products were in powder form.

## 4. Color of Meat Products

The pigments found in meat play a fundamental role in the perception of quality in meat and meat products. Myoglobin, the main pigment found in meat and meat products, is composed of two moieties: heme and globin protein. This pigment gives the characteristic bright cherry-red of fresh meat, which is perceived by consumers as an indicator of quality and freshness [80]. The relation between color and perceived quality and freshness is derived from the exposing conditions in retail where consumers can not perceive the odor, texture, and other sensory attributes before buying. Due to this important relation between color and quality at retail points, preserving the redness in meat and meat products is a crucial concern in retail point during storage [81].

Several factors can influence the myoglobin stability in meat and meat products: exposure to oxygen and UV-radiation, decay of product oxidative stability, pH and thermal treatment [80]. The oxidative state of iron and the ligand in the heme molecule are directly related to the color of meat and meat products, while the appreciated red color can be obtained in both oxymyoglobin and oxymyoglobin (ferrous state). Once an oxidizing agent interacts with myoglobin, the oxidative state of iron is modified from ferrous to ferric and yields the metmyoglobin, which gives the brown color to meat [2]. In this sense, preventing the progression of oxidative reactions can improve the stability and postpone the eventual decay of color.

In the face of this inevitable redness deterioration, the meat industry applies different strategies to improve the stability of redness during shelf life. One of the most common strategies is by modifying the myoglobin molecules with nitrite salts (in the case of dry-cured, fermented, and cooked sausages) [81]. The addition of nitrite salts in meat products is a common practice to improve the safety and shelf life of meat products in terms of oxidative stability [82]. Particularly for the antioxidant effect of nitrite, the mechanism is related to the stabilization of heme-iron. Iron is known to favor the lipid oxidation in meat products by catalyzing the formation of radicals once the heme iron is stabilized by forming the nitrosolmyoglobin pigment (dark red color) or the nitrosohemochrome (light and stable pink color) after heating. In both pigments, the iron is bonded to the porphyrin ring and does not catalyze the formation of radicals that can induce lipid and protein oxidation [83].

Recent experiments explored the influence of seed extract rich on polyphenols to improve the stability of color during storage (Table 3). One relevant source of antioxidants that can prevent the loss of color is the grape seed, as indicated by Aminzare et al. [84]. According to the authors, the cooked sausages produced with this natural extract in powder form had higher values of redness than control sausage (with nitrite) throughout the storage period (40 days at 4 °C). In the case of fermented and dry-cured sausages, grape extract was associated with the preservation of redness. According to Aquilani et al. [42], adding 10 g/kg of commercial grape seed extract (in powder form) led to the same values of redness than those observed in the control treatment (with nitrite) after ripening. Similarly, Pateiro et al. [85] observed the same protective effect on redness on a dry-cured sausage produced with 200 mg/kg of grape extract (freeze-dried hydroethanolic extract) during the ripening and storage period.

In the same line of thought, the polyphenols found in guarana seed can improve the preservation of redness during the storage of patties. According to Pateiro et al. [46], the highest concentration of guarana extract (1000 mg/kg; commercial extract in powder form) displayed the highest potential to prevent the formation of metmyoglobin in raw pork patties during 18 days at 2 °C. Moreover, the authors also indicated that the redness was better preserved than in control (without antioxidant). In a related experiment with guarana seed extract, Carvalho et al. [86] observed that grape seed extract affected the formation of metmyoglobin and the intensity of redness. The formation of the oxidized form of myoglobin was reduced and the redness was better preserved in patties produced with natural extract than in control and BHT treatments up to 12 days of refrigerated storage.

Peanuts skin is another relevant source of natural antioxidants that can improve the stability of color in patties. An experiment carried out with raw sheep patties obtained higher values of redness in samples containing peanut skin extract (obtained using a hydroethanolic solution and concentrated at low pressure) than in control treatment (without antioxidant) [87]. A similar outcome was reported in another experiment with cooked chicken patties [88]. The intensity of the patties produced with peanut skin was higher than those produced without control throughout the storage period. However, in both experiments, a reduction in redness gradually reduced was indicated during the progression of storage time. Although these studies indicate the protective effect of peanut skin against the gradual loss of redness, a different outcome was obtained from experiments with dry-cured sausage using this peanut skin hydroethanolic and concentrated extract [48] and with pork liver pâté [89], which did not influence the color in comparison to control treatments.

The preservation of red color was also reported for raw chicken meat added of hydroethanolic and concentrated acorn extract [59]. The authors evaluated the influence of acorn variety (holy, ordinary, and valonia) and indicated that ordinary and valonia varieties displayed the highest potentials to preserve redness during storage. Likewise, the incorporation of a commercial black rice extract (in the range of 0.4–1.2%) was associated with better preservation of redness in raw beef patties during six days at 2 °C [90].

It is also relevant to mention that some natural pigments can be co-extracted from seed matrix along with the polyphenols, which affect the color of the meat product. This outcome seems to explain the results obtained with a commercial cacao bean husk extract in the color of cooked pork sausage [93]. Conversely, *Euryale ferox* seed kernels extract (obtained using hydroethanolic solution followed by solvent evaporation at low pressure) did not affect the redness of cooked pork sausage [54].

In general, most of the seed extracts included in this review can improve the preservation of redness and delay the formation of oxymyoglobin. Increasing extract proportion in the formation of meat products does not necessarily improve the preservation of redness and can cause a reduction in redness at the beginning of storage period. This particular outcome was obtained using a freeze-dried pistachio seed hull extract in cooked chicken burger [94], commercial mustard seeds extract in raw beef meatballs [91], and with ground chia seeds extract (produced using hydroethanolic solution followed by the concentration at low pressure) in fresh pork sausage [92]. Since these studies indicated that seed extracts improved the antioxidant capacity of meat products and also delayed lipid oxidation, the effect seems to be explained by other factors such as the dilution with non-meat ingredients on fresh products before storage [95] and eventual low protection against myoglobin oxidation during storage.

Some extracts rich in particular antioxidants do not provide the same protection against myoglobin oxidation than others. This condition may be explained by their structure that eventually influences their antioxidant activity [96]. Another relevant condition is the formation of nitrosomyoglobin and nitrosohemochrome (cured meat products), which are more oxidatively stable than oxymyoglobin [83]. In this condition, natural antioxidants seem to have a minor role against color loss.

## 5. Lipid and Protein Oxidation

The oxidation of lipids and proteins play a central role in the oxidative stability of meat and meat products [97,98]. The lipid oxidation initiates by the exposure of unsaturated fatty acids to atmospheric oxygen (especially to the singlet oxygen) and additional energy (such as transition metals, high temperatures, and UV-radiation) that generates intermediate radical species. Eventually, these radicals will interact with unsaturated fatty acids, particularly in the double bounds by abstracting hydrogens and producing alkyl radicals [4,99].

Interactions between alkyl radicals with oxygen produce the peroxy radical. This radical will interact with other unsaturated fatty and generates a hydroperoxide and a new alkyl radical. The continuous formation of new alkyl radicals from fatty acids will propagate the oxidative degradation in the lipid phase. Moreover, the decomposition of hydroperoxides forms other radicals such as peroxy, hydroxyl, and alkoxyl radicals [4,99]. The accumulation of radicals and other non-reactive species favors the formation of low-reactive and stable molecules (such as carbonyl, alcohols, alkanes, and aldehydes) by transferring an atom or functional group [100]. Consequently, the oxidation of unsaturated fatty acids is terminated [4,99]. The progression of lipid oxidation can be examined and indicate the quality of fat components in the meat and meat products. First, primary products (such as hydroperoxides) from unsaturated fatty acids can be evaluated and, as the lipid oxidation continuous, the quantification of secondary products (malondialdehyde and aldehydes, for instance) can be assed [101,102].

In the case of protein oxidation, the reactions can take place in some of the main proteins of meat and meat products: myosin and troponin T. The oxidation seems to involve metals, heme proteins, and radical oxygen species (hydroxyl and hydroperoxyl radicals, for instance) that interact with amino acids (such as proline, lysine, methionine, and arginine) and form protein radicals [103]. Once these radicals are formed, the proteins in the proximity are modified: carbonyls are produced, sulfhydryl groups are lost, and protein cross-linking are formed [103,104].

Temperature plays an important role in the oxidative stability of lipids and proteins. Increasing temperature (such as during thermal processing of meat products) accelerate the oxidative reactions and favors the formation of oxidation products, which is a major concern in the storage of cooked meat and meat products [4,104].

Since lipid and protein oxidation share common intermediate radicals and prooxidant factors that are directly involved with the breakdown and modification of lipids and proteins, it is necessary to consider effective strategies to interrupt these oxidative reactions. In this sense, recent studies explored the potential antioxidant effect of seed extracts (Table 4). As indicated in the previous section, black rice is a rich source of polyphenols, especially anthocyanins. The use of black rice extract to prevent the progression of lipid oxidation was reported by Prommachart et al. [90] for raw beef patties during 6 days of refrigerated storage. According to the authors, the formation of lipid oxidation products was reduced in patties elaborated with commercial black rice extract, which also displayed a concentration-dependent effect (from 0.4% to 1.2% extract). In a similar way, the experiment performed by Loypimai et al. [105] indicated the same concentration-dependent effect on lipid oxidation of Sai Krok Isan (Thai fermented sausage) produced with black rice extract (in the range 0.2–1.0%) during four days of refrigerated storage. The formation of both hydroperoxides and TBARS were inhibited, especially at the highest concentration.

The grape seed extract is an interesting option to improve the oxidative stability of lipids in meat products. For instance, the experiment carried out by Tajik et al. [106] in raw buffalo patties revealed that lipid oxidation was delayed by a commercial grape seed extract (0.1% and 0.2%) in comparison to control treatment (without antioxidants) during nine days at 8 °C. In a similar way, grape seed extract slowed the formation of lipid oxidation products during the storage of cooked chicken sausage at 4 °C for 40 days [84]. In this case, it is important to notice that no prooxidative effect was indicated due to the simultaneous use of grape seed extract (0.08% and 0.16%) and sodium nitrite (100 mg/kg).

Another relevant outcome related to grape seed extract was reported by Aquilani et al. [42] in dry-fermented pork sausages. According to the authors, the sausages produced with natural extract displayed similar oxidative stability to control sausages produced with 30 ppm of sodium nitrite (0.93 mg vs 1.05 mg MDA/kg). However, the authors noticed an increased formation of volatile compounds formed from lipid oxidation. The antioxidant effect of grape seed extract was also observed in an experiment with dry-cured sausage [85]. While an increase in the formation of lipid oxidation products of control sausages (without antioxidant) was observed during ripening, the same outcome was not observed for sausages elaborated with BHT (200 mg/kg) and grape seed extract (regardless of concentration). During refrigerated storage, no increase in lipid oxidation levels was observed for any treatment.

The extracts obtained from guarana seeds were also associated with an antioxidant effect on meat products. In a recent experiment with raw pork patties, Pateiro et al. [46] observed that all three concentrations of guarana seed extract (250, 500, and 1000 mg/kg) were efficient to prevent the oxidative degradation of lipids during 18 days at 2 °C. The protection of proteins against the formation of carbonyls was also observed in patties produced with guarana seed extract in comparison to control (without antioxidant). In another experiment with raw lamb burgers, a similar protective effect on lipids and proteins was observed by the low oxidation indexes obtained from patties added of guarana seed extract up to 12 days of storage [86]. Another relevant outcome reported by the authors is the formation of volatile compounds derived from oxidative degradation of fatty acids. The content of many aldehydes, especially hexanal, was significantly lower than reported for control (without antioxidant).

Another source of polyphenols with potential applications in meat products is peanut skin. In raw sheep patties, the proanthocyanin-rich extract prevented the formation of both lipid and protein oxidation products in a similar way as reported for BHT treatment during 20 days of refrigerated storage [87]. A similar antioxidant was reported for cooked chicken patties [88]. In this experiment, the intense formation of lipid oxidation products was not observed in the treatment produced with peanut skin extract.

An interesting outcome was reported from the use of peanut skin extract produced in the Spanish *Salchichón* [48]. While the lipid oxidation was not influenced by the natural extract when compared with control (without antioxidant), a significant preservation of proteins (by reducing carbonyl formation) was observed on sausages produced with peanut skin extract. According to the authors, the encapsulation of n-3 fatty acids (susceptible to lipid oxidation) played an important role and restricted the contact between these fatty acids and antioxidant compounds mixed in the meat ass during processing. Although previous studies indicated the use of peanut skin is a relevant antioxidant for meat products, its use in pork liver pâté did not induce any change in the oxidative stability of lipids in comparison to control (with nitrite). Differently, the use of peanut skin extract in pork liver pâté did not influence the lipid oxidation and formation of derived volatile compounds [89].

Another related natural source of natural antioxidants among seeds is pistachio seeds [94]. The extract obtained from the hulls of pistachio seed inhibited the formation of lipid oxidation products incooked chicken burger. Although the TBARS values on day 0 were high (between 4.7 mg and 5.3 mg MDA/kg sample), the burgers produced with 5% and 7% of extract displayed lower oxidation levels in comparison to control (without antioxidant) during 14 days of refrigerated storage. Similarly, the use of lentil coat powder displayed antioxidant effect during the refrigerated storage of raw beef burgers (12 days at 4 °C) [107]. According to the authors, the antioxidant effect was similar to observed for BHA/BHT treatment (0.2%, 1:1) and dependent of the concentration of lentil powder applied (1%, 2%, and 3%) to the beef burger.

Buckwheat hull was indicated as a source of natural antioxidants with potential applications in meat products. A recent experiment carried out with cooked pork meatballs indicated that the freeze-dried aqueous extract obtained from buckwheat hulls delayed the formation of primary and secondary products of lipid oxidation in a similar fashion to observed for BHT (0.02%) [108]. A related experiment explored the use of cacao bean husk as a natural antioxidant for cooked pork sausage [93]. The authors indicated that the progression of lipid degradation via oxidative reactions was reduced in comparison to control (without antioxidant). Moreover, the authors noticed a concentration-dependent effect of cacao bean husk against lipid oxidation.

A recent experiment indicated that yellow, brown, and black mustard seeds (2.0%) can be applied in raw beef meatballs to prevent lipid oxidation [91]. According to the authors, the yellow mustard seeds had the highest antioxidant potential in comparison to brown and black mustard seeds during the refrigerated storage (15 days at 4 °C). The kernels obtained from *Euryale ferox* seed (500 mg/kg) were tested to prevent the oxidation of lipids in cooked pork sausage during 10 weeks [54]. The experiment carried out by Özünlü et al. [59] indicated that ground acorn (1000 mg phenolics/L) was effective against the progression of lipid oxidation in raw chicken meat. Another relevant experiment that supports the use of antioxidants found in seeds was conducted by Scapin et al. [92], who evaluated the influence of ground chia seed extract in fresh pork sausages during 28 days at 4 °C. According to the authors, the main antioxidant effect was observed in samples produced with 2.0% of extract.

Collectively, the oxidative reactions in lipids and proteins can be delayed by natural antioxidants found in seeds due to the evaluation in more than one type of meat products: dry-cured and fermented sausages during long processing periods and raw and cooked products during storage. This outcome is especially relevant for black rice [90,105], grape [42,84,85,106], guarana [46,86], and peanut skin extracts [48,87,88].

Regarding the potential to replace synthetic antioxidants (such as BHT, BHA, and nitrite salts) to slow lipid and protein oxidation during storage, recent studies support the use of polyphenols found in seeds as a suitable alternative. This outcome is supported by studies in raw burgers [87,107], cooked meatballs [108], and dry-cured sausage [85]. The concentration of extract in the formulation of meat product plays an important role to improve lipid oxidative stability as the most commonly evaluated variable in the recent studies displayed in Table 4.

## 6. Sensory Attributes

Sensory attributes are influenced by the progression of oxidative reactions that can take place during the processing and storage of meat and meat products. The accumulation of compounds associated with rancidity (due to formation of aldehydes, for instance), off-flavor, and off-odor as well as the modification of color (especially for the decay in redness) are the main effects derived from oxidative reactions in the sensory properties of meat and meat products [101,109].

The influence of antioxidant extracts from seeds in the sensory properties of meat products is shown in Table 5. An interesting source of natural antioxidants that can preserve the sensory properties of meat products is grape seeds. In a study with raw buffalo patties, Tajik et al. [106] indicated that samples produced with 0.1% of grape seed extract received higher scores than control (without antioxidant) patties for odor and overall acceptance. However, patties produced with both 0.1% and 0.2% of grape seed extract displayed lower values than the control for color acceptance after nine days at 4 °C.

In the case of cooked chicken sausage [84], grape seed extract (0.08% and 0.16%) did not significantly alter the acceptability of odor, color, and overall acceptance in relation to control treatment during 40 days of refrigerated storage. It is relevant to mention that control and experimental sausages were produced with sodium nitrite. In a similar way, in the experiment performed by Aquilani et al. [42], grape seed extract prevented the quality decay related to rancidity, off-flavors, and off-odor in dry-fermented pork sausages after ripening. However, this extract impacted the color uniformity and redness in comparison to control samples (with nitrite). In this study, only the control treatment was produced with nitrite.

Other extracts that can improve the preservation of sensory properties of meat products are cacao bean husk, peanut skin, and lentil coat powder. In the experiment with cacao bean husk extract [93], the effect of concentration (from 0.25% to 2%) on sensory attributes was tested in cooked pork sausage. The samples produced with 0.75% received higher scores than control (nitrite) for color, flavor, and overall acceptance. It is important to mention that all treatments included the addition of a nitrite salt. In the second case [87], the extract obtained from peanut skin improved the preservation of red color, superficial discoloration, and the overall acceptance of raw sheep patties during refrigerated storage (20 days at 2 °C) in comparison to control (without antioxidant). Moreover, the samples produced with the natural extract displayed similar or better results than those produced with BHT for the three attributes during storage. A similar effect was observed for the use of lentil coat powder in cooked beef burgers [107]. The three concentrations of lentil powder extract (1.0%, 2.0%, 3.0%) were efficient to slow the decay in acceptance of color and odor as well as in overall acceptability during 12 days at 4 °C. However, increasing the concentration of lentil extract induced a reduction in acceptance but with higher scores than those attributed to control (without antioxidant) samples.

Although some extracts display antioxidant potential, the effect on sensory properties may not be evident and even reduce the acceptance of some sensory attributes. This is the case of fresh pork sausage [92] produced with different levels of chia seed extract. According to the authors, non-significant differences were observed up to 2% of chia seed extract. Similarly, the addition of acorn extract in raw chicken meat [59] did not influence the sensory attributes during storage. Regarding the reduction of sensory acceptance, the acceptance is reduced in relation to control but still considered “acceptable” by panelists. This outcome was reported for yellow, brown, and black mustard seeds in raw beef meatballs [91] and pistachio seed hull in cooked chicken burger [94].

The sensory attributes of meat products produced with seed antioxidant extracts can preserve and delay the decrease in sensory quality of meat products during processing and storage. Especially for rancidity, off-flavor, discoloration, and loss of redness are delayed, which is in accordance with instrumental data that indicate a slow down on oxidative reactions on lipids, proteins, and pigments in meat (Table 3 and Table 4). However, an antagonist effect can be obtained due to the presence of natural colorants that may alter the color of the product as well as odorant and flavoring compounds extracted from seeds [91,94].

Finally, in the case of meat products produced with nitrite salts, the natural extracts play a second role as additional protection against the decay in sensory attributes when both (nitrite and natural extract) are added [84]. In the case that only the control treatment is produced with a nitrite salt, differences may be obtained in the color in comparison with other treatments with polyphenols [42]. In any case, a comparable effect can be obtained between meat products produced with a nitrite salt and another one with a natural extract (using an appropriate level of extract).

## 7. Conclusions

Natural antioxidants from seeds (extracted using optimized conditions and safe solvents) can improve the antioxidant capacity of meat and meat products and also prevent or delay the formation of radical species and oxidation products that reduces quality and shorten shelf life. The polyphenols found in seeds covered in the present review (caffeic acid, ferulic acid, (epi)catechin, procyanidin oligomers, quercetin, etc.) can improve the antioxidant capacity of meat products by preventing or delaying the oxidation of fatty acids, proteins, and meat pigments, which also resulted in the preservation of sensory attributes. Grape seed seems the most versatile source of natural antioxidants due to the appropriate preservation of raw, cooked, fermented, and dry-cured meat products. Other relevant sources are guarana, peanut, and black rice with antioxidant effects in more than one product, processing, or raw material.

## Figures and Tables

**Table 1:** Main phenolic compounds found in selected seeds.

Source	Main Polyphenols (Concentration)	Ref.
**CEREALS AND PSEUDO-CEREALS**		
Black rice (*Oryza sativa L.)*	Ferulic acid (26.1–215.2 μg/g), *p*-coumaric acid (6.6–28.5 μg/g), isoferulic acid (6.3–45.3 μg/g), cyanidin-3-glucoside (0.56–0.79 mg/g), and peonidin-3-glucoside (0.11–0.16 mg/g	[33]
	Ferulic acid (23.0–43.9 mg/100 g), vanillic acid (4.4–10.5 mg/100 g), and *p*-coumaric acid (2.0–6.6 mg/100 g)	[34]
Buckwheat (*Fagopyrum esculentum* Moench)	Quercetin-3-rutinoside (3.3–151.4 mg/100 g), isoorientin (0.1–2.7 mg/100 g), caffeic acid-pentoside (0.1–6.7 mg/100 g), procyanidin trimer (0.1–5.5 mg/100 g), and epiafzelechin-epicatechin (0.2–5.3 mg/100 g)	[35]
	1-*O*-Caffeoyl-6-*O*-rhamnopyranosyl-glycopyranoside (0.3–9.1 mg/100 g), epicatechin-gallate (25.4–91.3 mg/100 g), vitexin/isovitexin (101.6–188.8 mg/100 g), hyperin (53.5–274.1 mg/100 g), rutin (62.4–173.6 mg/100 g), and orientin/isorientin (8.4–26.3 mg/100 g)	[36]
Chia (*Salvia hispanica* L.)	Myricetin (7.0–1198.6 mg/g), quercetin (4.8–29.6 mg/g), protocatechuic acid (3.0–29.6 mg/g), and salicylic acid (6.2–241.2 mg/g)	[37]
	Rosmarinic acid (738.2 μg/g), caffeic acid (178.6 μg/g), quercetin (309.5 μg/g), and daidzein (110.5 μg/g)	[38]
**FRUITS**		
Cacao (*Theobroma cacao* L.)	Epicatechin (4.4–35.0 mg/g) and catechin (0.6–4.7mg/g)	[40]
	Epicatechin (7.0–15.8 mg/g) and catechin (1.0–6.2 mg/g)	[39]
Grape seed (*Vitis vinífera* L.)	Epicatechin gallate trimer (150.0–383.0 mg/g), epicatechin gallate (180.6 mg/g), and epicatechin (13.6 mg/g)	[42]
Guarana seed (*Paullinia cupana*)	Tyrosol (14.7 g/kg)	[46]
	Epicatechin (3.7–9.2 mg/g) and catechin (5.2–11.6 mg/g)	[44]
**PULSES AND NUTS**		
Lentil (*Lens culinaris*)	Catechin glucoside (2.2–6.6 mg/g), quercetin-*O*-pentoside (2.1–3.3 mg/g), prodelphinidin dimer (1.4–5.8 mg/g), and procyanidin dimer (1.6–4.3 mg/g)	[47]
Peanut skin (*Arachis hypogaea* L.)	Catechin (20.7 mg/100 g) and protocatechuic acid (3.8 mg/100 g)	[48]
	Proanthocyanidins (0.8–8.3 mg/100 g), di-*p*-coumaroyltartaric acid (13.8 mg/100 g), and *p*-coumaroylsinapoyltartaric acid (6.3 mg/100 g)	[49]
Pistachio (*Pistacia vera* L.)	Isoquercetin (27.3–578.2 mg/g), myrcitin-3-glucoside (62.7–75.3 mg/g), and quercetin-3-glucuronide (6.0–106.1 mg/g)	[50]
	Naringenin (28.1–49.9 μg/g), catechin (29.1–53.6 μg/g), and gallic acid (2.3–38.6 μg/g)	[51]
**CRUCIFEROUS VEGETABLES**		
Mustard (*Sinapis alba*)	Sinapine (10.2 mg/g), sinapoyl glucose (0.66 mg/g), and sinapic acid (0.19 mg/g)	[52]
**OTHER SOURCES**		
Acorn (*Quercus* spp.)	Gallic acid (3.6–417.5 μg/g), trigalloyl glucose (2.5–150.2 μg/g), trigalloyl-hexahydrodiphenoyl-glucose (1.4–240.8 μg/g), and valoneic acid dilactone (1.7–40.4 μg/g)	[53]
*Euryale ferox*	Gallic acid (107.5–392.2 mg/g), rutin (44.8–85.3 mg/g), and catechin (21.2–162.3 mg/g)	[54]

**Table 2:** Influence of extraction conditions in the phenolic content and antioxidant activity of seeds extracts.

Source	Extraction Conditions	Phenolic Content and Antioxidant Activity	Ref.
**CEREALS AND PSEUDOCEREALS**			
Black rice (*Oryza sativa* L.)	**Conventional extraction:** temperature (10, 30, and 50 °C); time (20, 50 and 80 min); and solid/solvent ratio (1 g/15, 30, and 45 mL)	TPC ^1^: 520.17 mg GAE ^2^/100 g; AA ^3^: 46.5% of DPPH ^4^ inhibition; optimum conditions: 34.7 °C, 80 min, 1 g/30 mL	[65]
	**Ultrasound:** frequency (35 kHz); temperature (30–60°C); pH (2–4); solvent (20–60% ethanol); and time (10–60 min)	TPC: 2124.98 mg GAE/100 g; optimum conditions: 36.0 °C, pH 2.5, 23.8% ethanol, and 22.9 min	[66]
Buckwheat (*Fagopyrum esculentum* Moench)	**Microwave irradiation:** time (15 min); solid/solvent ratio (1 g/50 mL); rotation (320 rpm); temperature (23–150 °C); and solvent (0, 50, and 100% ethanol)	TPC: 18.5 mg GAE/g; optimum conditions: 150 °C, 50% ethanol	[67]
Chia (*Salvia hispanica* L.)	**Conventional extraction:** solid/solvent ratio (1 g/10 mL); solvent (0, 20, 50, 80, and 100% ethanol); extraction cycles (1–4); and time (1–72 h)	TPC: 42% increase; optimum conditions: 50% ethanol, and 4 cycles of 1 h each	[68]
**FRUITS**			
Cacao bean shell (*Theobroma cacao* L.)	**Pressurized liquid extraction:** solid/solvent ratio (1 g/3 mL); pressure (10.35 MPa); time (5, 30; 50 min); and temperature (60–90 °C)	Increasing time and temperature increased the extraction of flavonols (90 °C, 50 min) and antioxidant activity (DPPH and FRAP ^5^ assays); optimum conditions: 90 °C and 50 min for polyphenols; and 75 °C and 50 min for antioxidant activity	[69]
Grape seed (*Vitis vinífera* L.)	**Ultrasound:** frequency (20 kHz); power (150 W); time (15 min); temperature (<30 °C); and solvent (methanol); **Soxhlet:** temperature (RT ^6^); time (12 h); solvent (methanol)	TPC: 104.19 mg GAE/g; AA: 109.3 aTocE ^7^/g for DPPH radical assay; defatted seeds with ultrasound followed by Soxhlet extraction of polyphenols	[70]
	**Microwave irradiation:** time (15 min); solid/solvent ratio (1 g/10–50 mL); solvent (10–90% ethanol); time (2-32 min); and temperature (40–60 °C)	TPC: 96.3 mg GAE/g; optimum conditions: 47.2% ethanol, 1 g/45.3 mL, ratio, 4.6 min	[71]
Guarana seed (*Paullinia cupana*)	**Conventional extraction (cold):** solid/solvent ratio (1 g/3 g); temperature (25 °C); time (24 h); particle size (25, 125, and 1680 μm); and solvent (0, 50, 65, 80, and 100% ethanol)	Highest TPC was obtained with 1680 μm particles and 50% ethanol solution	[72]
	**Conventional extraction (hot):** solid/solvent ratio (1 g/3 g); agitation (48 rpm); temperature (40, 50, and 60 °C); and time (1–6 h)	Highest TPC was obtained after 6 h, no effect of temperature (40–60 °C)	[72]
	**Enzyme-assisted extraction:** solid/solvent ratio (1 g/3 g); time (4 h); agitation (48 rpm); pH (4.8); time (4 h); enzyme (pectinase:celulase, 1:0, 1:1, 0:1); and temperature (40 and 50 °C)	Highest TPC was obtained using pectinase alone 50 °C	[72]
	**Super-critical CO_2_:** flow (6 mL/min); pressure (100, 200, and 300 bar); temperature (40, 50, and 60 °C); co-solvent (0, 10, 20, 40% of methanol, ethanol, and their 1:1 combination); and time (20, 40, and 60 min)	TPC: 105.76 mg PE ^8^/g; optimum conditions: 300 bar, 40 °C, 40% of 1:1 ethanol:methanol, and 40 min	[73]
**NUTS**			
Peanut skin (*Arachis hypogaea* L.)	**Conventional extraction:** time (5–150 min); temperature (25–90 °C); and solvent (20–100% ethanol)	TPC: 0.39 g GAE/g; AA: 79.9% of inhibition in the DPPH radical assay and IC_50_ of 0.26 μg/mL in the ABTS ^9^ radical assay; optimum conditions: 71.6 °C and 74% ethanol	[74]
	**Sub-critical water:** temperature (140, 180 and 220 °C); flow (3, 5, and 7 g/min); and solvent (0, 50 and 95% ethanol)	TPC: 136.9 mg/g; AA: IC_50_ of 10.5 μg/mL in DPPH radical assay and IC_50_ of 17.05 μg/mL optimum conditions: 220 °C, 7 g/min, and 60.5% ethanol	[75]
Pistachio (*Pistacia vera* L.)	**Sub-critical water:** solid/solvent ratio (1 g/25 mL); pressure (6.9 MPa); flow (4 mL/min); and temperature (110–190 °C)	TPC: 39.5 and 39.4 g/kg at 150 and 170 °C, respectively; AA: ABTS radical (1.18 mmol TE ^10^/g), DPPH radical (0.84 mmol TE/g) and FRAP (1.20 mmol TE/g) assays at 190 °C	[76]
	**Ultrasound:** solid/solvent ratio (1 g/60 mL); and solvent (methanol:water:formic acid; 80:19:1)	TPC of 81.8 g/kg; AA: 0.47, 0.51, and 0.49 mmol TE/g for ABTS, DPPH, and FRAP assays, respectively	[76]
**OTHER SOURCES**			
Acorn (*Quercus* spp.)	**Eco-friendly extraction:** solid/solvent ratio (1 g/50 mL); time (10 min); solvent (water:glycerol:CD; 40–100%:0–60%:1–13%); and temperature (40, 60, and 80 °C)	TPC: 122.2 mg/g; AA: DPPH radical (1209.8 μmol TE/g) and reducing power (555.8 µmol AAE ^11^/g) assays optimum conditions: water: glycerol: CD ^12^, 27:60:13 and 80 °C	[77]

^1^ TPC: total phenolic content; ^2^ GAE: gallic acid equivalent; ^3^ AA: antioxidant activity; ^4^ DPPH: 2,2-diphenyl-1-picrylhydrazyl; ^5^ FRAP: ferric reducing ability of plasma; ^6^ RT: room temperature; ^7^ aTocE: alpha-tocopherol equivalent; ^8^ PE: pyrogallol equivalent; ^9^ ABTS: 2,2’-azino-bis-(3-ethyl-benzothiazoline-6-sulphonic acid); ^10^ TE: trolox equivalent; ^11^ AAE: ascorbic acid equivalent; and ^12^ CD: 2-hydroxypropyl-β-cyclodextrin.

**Table 3:** Influence of seed extracts on the color of meat products.

Source and Concentration	Meat Product	Sampling Point or Storage Conditions	Effect on Color	Ref.
Grape seed (0.08 and 0.16%)	Cooked chicken sausage	40 days at 4 °C	Improved the preservation of redness	[84]
Grape seed (10 g/kg)	Dry-fermented pork sausages	After ripening	Similar to control with nitrite	[42]
Grape seed (50, 200 and 1000 mg/kg)	Dry-cured sausage	During ripening and during 7 months at 4 °C	Effect was dependent of extract concentration; higher redness values were obtained from 200 mg/kg treatment	[85]
Guarana seed (250, 500, and 1000 mg/kg)	Raw pork patty	18 days at 2 °C	Improved the preservation of redness and reduced the formation of metmyoglobin (1000 mg/kg)	[46]
Guarana seed (250 mg/kg)	Raw lamb burgers	18 days at 2 °C	Slowed the reduction of redness and formation of metmyoglobin	[86]
Peanut skin (1000 mg/kg)	Raw sheep patties	20 days at 2 °C	Improved the stability of redness	[87]
Peanut skin (70 mg GAE^1^/kg)	Cooked chicken patties	15 days at 1 °C	Reduced the loss of redness	[88]
Peanut skin (2000 mg/kg)	Dry-cured sausage (Spanish *salchichón*)	After ripening	Not significant effect	[48]
Peanut skin (1000 mg/kg)	Pork liver pâté	60 days at 4 °C	Slight differences	[89]
Acorn (1000 mg phenolics/L)	Raw chicken meat	14 days at 2 °C	Preservation of redness	[59]
Black rice (0.4%, 0.8%, and 1.2%)	Raw beef patties	6 days at 2 °C	Reduced the loss of redness	[90]
*Euryale ferox* seed kernels (500 mg/kg)	Cooked pork sausage	10 weeks at 8 °C	Not significant effect on redness	[54]
Yellow, brown, and black mustard seeds (2.0%)	Raw beef meatballs	15 days at 4 °C	Reduced redness	[91]
Chia seed (1.0%, 1.5%, and 2.0%)	Fresh pork sausage	28 days at 4 °C	Lower redness than control	[92]

^1^ GAE: gallic acid equivalent.

**Table 4:** Influence of seed extracts on the lipid and protein oxidation of meat products.

Source	Meat Product	Sampling Point or Storage Conditions	Effect on Lipid and Protein Oxidation	Ref.
Black rice (0.4%, 0.8%, and 1.2%)	Raw beef patties	Six days at 2 °C	All extract inhibited the lipid oxidation	[90]
Black rice (0.2–1.0 g/100 g)	Sai Krok Isan	Four days at 4 °C	Reduced the formation of peroxides and TBARS^1^	[105]
Grape seed (0.1% and 0.2%)	Raw buffalo patties	Nine days at 8 °C	All extracts inhibited lipid oxidation	[106]
Grape seed (0.08% and 0.16%)	Cooked chicken sausage	40 days at 4 °C	Lower values than control	[84]
Grape seed (10 g/kg)	Dry-fermented pork sausages	After ripening	Similar antioxidant effect to sodium nitrite; higher formation of volatile compounds from lipid oxidation	[42]
Grape seed (50, 200, and 1000 mg/kg)	Dry-cured sausage	During ripening and during 7 months at 4 °C	Inhibited lipid oxidation during both ripening and storage periods	[85]
Guarana seed (250, 500, and 1000 mg/kg)	Raw pork patties	18 days at 2 °C	All extract reduced the formation of carbonyls and TBARS	[46]
Guarana seed (250 mg/kg)	Raw lamb burgers	18 days at 2 °C	Slowed lipid and protein oxidation up to 12 days; inhibited the formation of volatile aldehydes	[86]
Peanut skin (1000 mg/kg)	Raw sheep patties	20 days at 2 °C	Slowed both lipid and protein oxidation	[87]
Peanut skin (70 mg GAE^2^/kg)	Cooked chicken patties	15 days at 1 °C	Inhibited lipid oxidation	[88]
Peanut skin (2000 mg/kg)	Dry-cured sausage (Spanish *salchichón*)	After ripening	No effect on lipid oxidation; reduction of protein oxidation	[48]
Peanut skin (1000 mg/kg)	Pork liver pâté	160 days at 4 °C	Not significant effect	[89]
Pistachio seed hull (2.0%, 5.0%, and 7.0%)	Cooked chicken burger	10 days 4 °C	Lowest TBARS values sing 5 and 7%	[94]
Lentil coat powder (1.0, 2.0 and 3.0%)	Raw beef burgers	12 days at 4 °C	Slowed the evolution of lipid oxidation	[107]
Buckwheat hulls (0.5%)	Cooked pork meatballs	180 days at −18 °C	Reduced the formation of peroxides and TBARS	[108]
Cacao bean husk (0.25%, 0.5%, 1.0%, and 2.0%)	Cooked pork sausage	Seven days at 4 °C	Inhibition in a concentration-dependent manner	[93]
*Euryale ferox* seed kernels (500 mg/kg)	Cooked pork sausage	10 weeks at 8 °C	Inhibited lipid oxidation throughout storage	[54]
Acorn (1000 mg phenolics/L)	Raw chicken meat	14 days at 2 °C	All extracts reduced lipid and protein oxidation	[59]
Yellow, brown, and black mustard seeds (2.0%)	Raw beef meatballs	15 days at 4 °C	All powders slowed lipid oxidation	[91]
Chia seeds (1.0%, 1.5%, and 2.0%)	Fresh pork sausage	During 28 days at 4 °C	Slowed the generation of lipid oxidation products	[92]

^1^ TBARS, thiobarbituric acid-reactive substances; ^2^ GAE: gallic acid equivalent.

**Table 5:** Influence of seed extracts on the sensory attributes of meat products.

Source	Meat Product	Sampling Point or Storage Conditions	Effect on Sensory Attributes	Ref.
Grape seed (0.1% and 0.2%)	Raw buffalo patties	After nine days at 8 °C	0.1% extract preserved the odor and overall acceptance	[106]
Grape seed (0.08% and 0.16%)	Cooked chicken sausage	After cooking	Similar scores for odor, color and overall acceptance	[84]
Grape seed (10 g/kg)	Dry-fermented pork sausages	After ripening	Lower acceptance for color uniformity, redness than control; rancidity was not perceived	[42]
Cacao bean husk (0.25%, 0.5%, 0.75%, 1.0%, and 2.0%)	Cooked pork sausage	After cooking	Preserved color, flavor and overall acceptance; optimum concentration: 0.75%	[93]
Peanut skin (1000 mg/kg)	Raw sheep patties	20 days at 2 °C	Extended the acceptance of red color, reduced superficial discoloration and formation of off-odor	[87]
Lentil coat powder (1.0%, 2.0%, and 3.0%)	Cooked beef burgers	12 days at 4 °C	Improved the preservation of color, odor and overall acceptability; negative influence of concentration	[107]
Acorn (1000 mg phenolics/L)	Raw chicken meat	14 days at 2 °C	Not significant effect on color acceptance	[59]
Chia seed (1.0%, 1.5%, and 2.0%)	Fresh pork sausage	After processing	Similar scores to control with antioxidant for appearance, color, odor and intention to purchase; slightly effect in taste (2%)	[92]
Yellow, brown, and black mustard seeds (2.0%)	Raw beef meatballs	15 days at 4 °C	Decreased during storage; yellow mustard burgers received the highest scores	[91]
Pistachio seed hull (2.0%, 5.0%, and 7.0%)	Cooked chicken burger	After cooking	Reduced acceptance of color (5 and 7%)	[94]
